# Long-term weight change and risk of breast cancer in the European Prospective Investigation into Cancer and Nutrition (EPIC) study

**DOI:** 10.1093/ije/dyab032

**Published:** 2021-03-23

**Authors:** Merete Ellingjord-Dale, Sofia Christakoudi, Elisabete Weiderpass, Salvatore Panico, Laure Dossus, Anja Olsen, Anne Tjønneland, Rudolf Kaaks, Matthias B Schulze, Giovanna Masala, Inger T Gram, Guri Skeie, Ann H Rosendahl, Malin Sund, Tim Key, Pietro Ferrari, Marc Gunter, Alicia K Heath, Konstantinos K Tsilidis, Elio Riboli, additional authors

**Affiliations:** 1 Department of Epidemiology and Biostatistics, School of Public Health, Imperial College London, UK; 2 MRC Centre for Transplantation, King’s College London, London, UK; 3 International Agency for Research on Cancer, World Health Organization, Lyon, France; 4 Department of Clinical and Experimental Medicine, University of Naples Frederico II, Naples, Italy; 5 Nutrition and Metabolism Section, International Agency for Research on Cancer, World Health Organization, Lyon, France; 6 Department of Public Health, Aarhus University, Aarhus, Denmark; 7 Danish Cancer Society, Research Center, Copenhagen, Denmark; 8 Institute of Public Health, University of Copenhagen, Copenhagen, Denmark; 9 Division of Cancer Epidemiology, German Cancer Research Center (DKFZ), Heidelberg, Germany; 10 Department of Molecular Epidemiology, German Institute of Human Nutrition Potsdam-Rehbruecke, Nuthetal, Germany; 11 Institute of Nutritional Science, University of Potsdam, Potsdam, Germany; 12 Cancer Risk Factors and Life-Style Epidemiology Unit, Institute for Cancer Research, Prevention and Clinical Network—ISPRO, Florence, Italy; 13 Department of Community Medicine, Faculty of Health Sciences, University of Tromsø, The Arctic University of Norway, Tromsø, Norway; 14 Department of Clinical Sciences Lund, Division of Oncology, Lund University and Skåne University Hospital, Lund, Sweden; 15 Department of Surgical and Perioperative Sciences, Umeå University, Umeå, Sweden; 16 Cancer Epidemiology Unit, Nuffield, Department of Population Health, University of Oxford, Oxford, UK; 17 Department of Hygiene and Epidemiology, School of Medicine, University of Ioannina, Ioannina, Greece

**Keywords:** long-term weight change, breast cancer, cohort study

## Abstract

**Background:**

The role of obesity and weight change in breast-cancer development is complex and incompletely understood. We investigated long-term weight change and breast-cancer risk by body mass index (BMI) at age 20 years, menopausal status, hormone replacement therapy (HRT) and hormone-receptor status.

**Methods:**

Using data on weight collected at three different time points from women who participated in the European Prospective Investigation into Cancer and Nutrition (EPIC) study, we investigated the association between weight change from age 20 years until middle adulthood and risk of breast cancer.

**Results:**

In total, 150 257 women with a median age of 51 years at cohort entry were followed for an average of 14 years (standard deviation = 3.9) during which 6532 breast-cancer cases occurred. Compared with women with stable weight (±2.5 kg), long-term weight gain >10 kg was positively associated with postmenopausal breast-cancer risk in women who were lean at age 20 [hazard ratio (HR) = 1.42; 95% confidence interval 1.22–1.65] in ever HRT users (HR = 1.23; 1.04–1.44), in never HRT users (HR = 1.40; 1.16–1.68) and in oestrogen-and-progesterone-receptor-positive (ER+PR+) breast cancer (HR = 1.46; 1.15–1.85).

**Conclusion:**

Long-term weight gain was positively associated with postmenopausal breast cancer in women who were lean at age 20, both in HRT ever users and non-users, and hormone-receptor-positive breast cancer.

Key Messages

- Long-term weight gain was positively associated with postmenopausal breast cancer in women who were lean at age 20 years, in hormone replacement therapy ever users and non-users.
- Long-term weight gain was positively associated with hormone-receptor-positive breast cancer.
- It is important to avoid weight gain from young to middle adulthood for the prevention of postmenopausal breast cancer.

## Introduction

Obesity in middle adulthood has been associated with increased breast-cancer risk in postmenopausal women,^1–3^ but adiposity from early life to menopause has been inversely associated with postmenopausal breast-cancer incidence.^4^^,^^5^ Studies have reported that weight gain before age 30 years is associated with lower breast-cancer risk in both pre- and postmenopausal women^6^^,^^7^ whereas weight gain in middle adulthood has been associated with an increased risk of postmenopausal but not premenopausal breast cancer.^7^ Weight loss after menopause has been associated with reduced breast-cancer risk.^8^^,^^9^

Weight change and breast cancer were previously investigated in the European Prospective Investigation into Cancer and Nutrition (EPIC) study with 5.8 years’ follow-up.^10^ It reported that weight gain (>20 kg) between age 20 years and cohort entry was associated with increased postmenopausal breast-cancer risk,^10^ restricted to non-users of hormone replacement therapy (HRT) [hazard ratio (HR) = 1.52; 95% confidence interval 1.08–2.13]. There was no association between weight gain and premenopausal breast cancer. A subsequent analysis in the EPIC-PANACEA study^11^ on weight change in middle adulthood found evidence for a positive association between high weight gain (0.83–4.98 kg/year) and premenopausal breast cancer (HR = 1.37; 1.02–1.85), but no support for an association with postmenopausal breast-cancer risk.^11^

With longer follow-up and more breast-cancer cases, we re-evaluated the association between long-term weight change and breast-cancer risk by investigating whether body mass index (BMI) at age 20 years or HRT use modified this association, and whether there were differences according to tumour hormone-receptor status or menopausal status at diagnosis.

## Methods

### Study population

The EPIC study is a large prospective cohort including >520 000 adult participants (aged 20–99 years) from 10 European countries.^12^ Medical history and dietary and lifestyle questionnaires were self-administered and anthropometric measurements and blood samples obtained at enrolment (1992–2000). All participants gave their written informed consents. Ethical approval was obtained from the International Agency for Research on Cancer ethical review board and from local ethical committees of the EPIC centres. Since the present study included analysing weight at age 20 years, which was only available for Italy, the UK, Germany, Sweden, Denmark and Norway, the data set was restricted to those countries. A flow chart detailing the study population is provided as Supplementary Figure 1, available as Supplementary data at *IJE* online.

### Assessment of weight change

Weight at cohort entry was assessed by trained staff for all countries except Norway, which collected self-reported weight and height, and the UK, where weight and height were either measured or self-reported. All countries collected self-reported weight at age 20 years and weight at follow-up. Follow-up was on average 14.2 years after baseline in the current study. Weight change was calculated as the difference between weight at cohort entry and weight at age 20 years using the difference between weight at follow-up and weight at age 20 years as an update. Weight change was categorized^10^ as follows: <–2.5, –2.5/+2.5 (reference), 2.51–5, 5.1–10 and >10 kg. Participants with missing data for weight at age 20 years were excluded.

### Assessment of menopausal status

Menopausal status at cohort entry was defined as follows: women who reported fewer than four menses in the past year or a bilateral ovariectomy were considered postmenopausal. The rest of the women were considered pre/perimenopausal. In case of incomplete or missing questionnaire data, women <55 years old at cohort entry were considered pre/perimenopausal and women >55 years old were defined as postmenopausal.

### Assessment of end point

Incident breast-cancer cases were identified through population cancer registries or by active follow-up, including health-insurance records, cancer and pathology registries and contact with participants and their next-of-kin. Censoring dates depended on the dates at which cancer registries in each centre were considered complete. Women were followed up from cohort entry until diagnosis of the first incident cancer, death, emigration or end of follow-up, whichever occurred first. Cancer cases were classified according to the International Classification of Diseases for Oncology 2nd Revision (ICD-O-2) and breast cancer was defined as C50 (C50.0–C50.9). Information on hormone-receptor status (i.e. oestrogen receptor, progesterone receptor) was available from pathology reports after 1997. These were classified as oestrogen-receptor-positive (ER+), oestrogen-receptor-negative (ER–), ER+ progesterone-receptor-positive (ER+PR+) and ER– progesterone-receptor-negative (ER–PR–) breast cancer. Since no data were available on menopausal status at diagnosis, age at diagnosis was used as a proxy. Premenopausal breast cancer was defined as having a breast-cancer diagnosis at age <55 years and postmenopausal breast cancer at age ≥55 years. Premenopausal breast cancer was studied in women who were pre- or perimenopausal at cohort entry (referred to as ‘premenopausal’) and postmenopausal breast cancer in all women.

### Statistical analyses

Cox proportional-hazard models with time-dependent covariates were used to estimate HRs and 95% confidence intervals with age as the timescale. For women with no follow-up information on weight, entry time was defined as age at cohort entry and exit time as age at cancer diagnosis or censoring. For women with information from a follow-up visit, we created two separate records for cohort entry and follow-up, accommodating the corresponding information on the time-dependent covariates. For the cohort-entry record, entry time was age at cohort entry, exit time was age on the day before the follow-up visit and the status was non-case. For the follow-up record, entry time was age at the follow-up visit and exit time was age at cancer diagnosis or censoring. All analyses were stratified by study centre and age at cohort entry (5-year categories).

Models were adjusted for the following lifestyle characteristics collected at cohort entry: weight at age 20 years, height, age at menarche, age at first birth, number of full-term pregnancies, education, alcohol consumption, smoking status, physical activity, oral-contraceptive use and HRT use (the categories are shown in Table 1). For measuring physical activity, we used the Combined Physical Activity Index based on cross-tabulation of occupational activity by non-working activities cycling and sports activities.^13^

**Table 1:** Frequency distribution and mean values of covariates by weight change from age 20 years to baseline (*n* = 150 257)

	Weight change in kilograms (kg)					
Characteristics	<–2.5 (*n* = 12 313)	±2.5 (*n* = 23 312)	2.5–5 (*n* = 17 520)	5.1–10 (*n* = 33 960)	10+ (*n* = 63 152)	Total (*n* = 150 257)
			**Mean (s.d.)**			
Age at recruitment (years)	48.7 (12.4)	45.9 (12.6)	48.7 (10.8)	50.8 (9.7)	53.3 (8.4)	50.7 (10.5)
Weight aged 20 years (kg)	64.6 (10.1)	57.9 (7.2)	56.6 (6.9)	56 (6.9)	56.1 (7.9)	57.1 (8)
Height (cm)	163.8 (6.4)	164 (6.4)	163.8 (6.3)	163.6 (6.4)	163.7 (6.5)	163.7 (6.4)
			***n* (%)**			
**Age at menarche (years)**						
<12	1699 (13.8)	2964 (12.7)	2133 (12.2)	4355 (12.8)	8931 (14.1)	20 082
12	2287 (18.6)	4390 (18.8)	3333 (19)	6262 (18.4)	11 919 (18.9)	28 191
13	3208 (26.1)	6380 (27.4)	4711 (26.9)	8766 (25.8)	15 371 (24.3)	38 436
14	2616 (21.2)	5227 (22.4)	3897 (22.2)	7873 (23.2)	14 069 (22.3)	33 682
>14	2240 (18.2)	3943 (16.9)	3082 (17.6)	5973 (17.6)	11 470 (18.2)	26 708
Missing	263 (2.1)	408 (1.8)	364 (2.1)	731 (2.2)	1392 (2.2)	3158
**Age at first birth (years)**						
<20	750 (6.1)	1320 (5.7)	1211 (6.9)	2759 (8.1)	6791 (10.8)	12 831
20–30	6756 (54.9)	12 721 (54.6)	10 864 (62)	22 750 (67)	43 901 (69.5)	96 992
>30	1126 (9.1)	2128 (9.1)	1702 (9.7)	2873 (8.5)	4712 (7.5)	12 541
Nulliparous	3474 (28.2)	6761 (29)	3474 (19.8)	5106 (15)	6950 (11)	25 765
Missing	207 (1.7)	382 (1.6)	269 (1.5)	472 (1.4)	798 (1.3)	2128
**Number of full-term pregnancies**						
Nulliparous	3474 (28.2)	6761 (29)	3474 (19.8)	5106 (15)	6950 (11)	25 765
1	2014 (16.4)	3531 (15.2)	2788 (15.9)	5378 (15.8)	9612 (15.2)	23 323
2	4176 (33.9)	8202 (35.2)	7057 (40.3)	14 515 (42.7)	27 130 (43)	61 080
3	1842 (15)	3329 (14.3)	3038 (17.3)	6477 (19.1)	13 416 (21.2)	28 102
4	470 (3.8)	886 (3.8)	726 (4.1)	1649 (4.9)	3999 (6.3)	7730
5	118 (1)	204 (0.9)	144 (0.8)	326 (1)	1022 (1.6)	1814
>5	42 (0.3)	64 (0.3)	59 (0.3)	120 (0.4)	439 (0.7)	724
Missing	177 (1.4)	335 (1.4)	234 (1.3)	389 (1.2)	584 (0.9)	1719
**Education**						
None	63 (0.5)	94 (0.4)	61 (0.4)	174 (0.5)	445 (0.7)	837
Primary school	2376 (19.3)	3217 (13.8)	2950 (16.8)	7012 (20.7)	18 730 (29.7)	34 285
Technical/professional school	3629 (29.5)	6670 (28.6)	5645 (32.2)	11 567 (34.1)	22 113 (35)	49 624
Secondary school	1966 (16)	4440 (19)	2988 (17.1)	5621 (16.5)	8871 (14.1)	23 886
University degree	3386 (27.5)	7359 (31.6)	4782 (27.3)	7346 (21.6)	9245 (14.6)	32 118
Missing	893 (7.2)	1532 (6.6)	1094 (6.2)	2240 (6.6)	3748 (5.9)	9507
**Alcohol consumption (glass/day)**						
Non-drinkers	190 (1.5)	237 (1.0)	171 (1.0)	2172 (1.5)	1168 (1.9)	2172
1 glass/day	9566 (77.7)	17 927 (76.9)	13 570 (77.5)	11 5848 (77.1)	48 804 (77.3)	11 5848
>1 glass/day	2417 (19.6)	4970 (21.3)	3580 (20.40)	30 529 (20.3)	12 405 (19.6)	30 529
Missing	140 (1.1)	178 (0.8)	199 (1.1)	1708 (1.1)	775 (1.2)	1708
**Smoking status^a^**						
Never	5521 (44.8)	12 325 (52.9)	8750 (49.9)	16 802 (49.5)	30 173 (47.8)	73 571
Former	2648 (21.5)	4968 (21.3)	4308 (24.6)	8616 (25.4)	17 328 (27.4)	37 868
Current	3703 (30.1)	5290 (22.7)	3874 (22.1)	7365 (21.7)	13 447 (21.3)	33 679
Missing	441 (3.6)	729 (3.1)	588 (3.4)	1177 (3.5)	2204 (3.5)	5139
**Physical activity^b^**						
Inactive	2109 (17.1)	3383 (14.5)	2649 (15.1)	5598 (16.5)	13 334 (21.1)	27 073
Moderately inactive	3668 (29.8)	7411 (31.8)	5551 (31.7)	10 914 (32.1)	21 106 (33.4)	48 650
Moderately active	3697 (30)	7414 (31.8)	5757 (32.9)	10 877 (32)	17 844 (28.3)	45 589
Active	2620 (21.3)	4778 (20.5)	3269 (18.7)	5924 (17.4)	9686 (15.3)	26 277
Missing	219 (1.8)	326 (1.4)	294 (1.7)	647 (1.9)	1182 (1.9)	2668
**Oral-contraceptive use**						
Never	4484 (36.4)	7155 (30.7)	5540 (31.6)	11 866 (34.9)	25 906 (41)	54 951
Ever	7686 (62.4)	15 937 (68.4)	11 824 (67.5)	21 683 (63.9)	36 531 (57.9)	93 661
Missing	143 (1.2)	220 (0.9)	156 (0.9)	411 (1.2)	715 (1.1)	1645
**Menopausal status at baseline**						
Premenopausal	5221 (42.4)	11 760 (50.5)	5540 (31.6)	11 473 (33.8)	14 279 (22.6)	50 122
Perimenopausal	1802 (14.6)	3446 (14.8)	11 824 (67.5)	6691 (19.7)	13 367 (21.2)	28 384
Postmenopausal	5290 (43)	8106 (34.8)	156 (0.9)	15 796 (46.5)	35 506 (56.2)	71 751
**Hormone replacement therapy**						
Never	8740 (71)	17 135 (73.5)	11 832 (67.5)	21 556 (63.5)	37 695 (59.7)	96 958
Ever	2819 (22.9)	4783 (20.5)	4361 (24.9)	9907 (29.2)	20 602 (32.6)	42 472
Missing	754 (6.1)	1394 (6.0)	1327 (7.6)	2497 (7.4)	4855 (7.7)	10 827
						

a Never/former (quit ≤10, 11–20 and >20 years ago)/current (current and pipe/cigar smoking, current and 1–15, current and 16–25, and >26 cigarettes/day, missing).

b Combined Physical Activity Index—using cut-points determined in Cambridge in a heart-rate-monitoring validation study and categorizes the population into four activity levels based on a cross-tabulation of occupational activity in four categories by cycling and sports activities (aerobics, swimming and jogging).

### Cross-classification

We divided BMI at age 20 years (BMI_20_) into BMI_20_ <25 and ≥25 kg/m². For the cross-classification on weight change and BMI_20_, we generated a cross-classification variable joining together the indicators of the groups of weight change and BMI_20_ and ran the same model with two different reference categories: Reference 1 (BMI_20_ <25 kg/m² and ±2.5 kg) and Reference 2 (BMI_20_ ≥25 kg/m² and ±2.5 kg). We presented estimates for BMI_20_ ≥25 kg/m² with both reference categories to examine whether the smaller effect of weight gain for BMI_20_ ≥25 kg/m² was due to an already higher risk in this group compared with BMI_20_ <25 kg/m². For the cross-classification on weight change, BMI_20_ and menopausal hormone therapy, we generated a cross-classification variable pasting together the indicators of the groups for weight change, BMI_20_ and HRT, and ran the model with four different reference groups: Reference 1 (BMI_20_ <25 kg/m², ±2.5 kg in non-users); Reference 2 (BMI_20_ ≥25 kg/m², ±2.5 kg in non-users); Reference 3 (BMI_20_ <25 kg/m², ±2.5 kg in HRT users); Reference 4 (BMI_20_ ≥25 kg/m², ±2.5 kg in HRT users).

The *p*-value for the interaction term was obtained using the likelihood-ratio test comparing a cross-classification model with a model without the cross-classification. A test for linear trend (*p*-trend) was performed by fitting ordinal values corresponding to exposure categories and testing whether the slope coefficient differed from zero. A two tailed *p*-value of <0.05 was considered statistically significant. All statistical analyses were performed using Stata 13.1 (StataCorp, College Station, TX, USA).

## Results

In total, 150 257 women with a median age of 51 years (age range 20–99) at cohort entry were followed for an average of 14 years (standard deviation = 3.9 and a total of 2 133 649 person-years) (Supplementary Table 1, available as Supplementary data at *IJE* online). At cohort entry, 78 506 (52.3%) were pre- or perimenopausal and 71 751 (47.7%) were postmenopausal. Follow-up weight was available for 92 047 women. During follow-up, there were 6352 incident breast-cancer cases overall, which included 1461 premenopausal breast-cancer cases in women who were pre-/perimenopausal at cohort entry, 1388 postmenopausal breast-cancer cases in women who were pre-/perimenopausal at entry and 3503 postmenopausal breast-cancer cases in women who were postmenopausal at cohort entry (Supplementary Figure 1, available as Supplementary data at *IJE* online).

More than 50% of the women from Italy, Denmark and Sweden gained >10 kg from age 20 years to cohort entry. The frequency of weight loss was highest in the UK (11.2%) and between 6.1% and 7.3% in other centres. We observed the same pattern of weight distribution by country for both pre- and postmenopausal women, although more postmenopausal women gained >10 kg (Supplementary Table 2, available as Supplementary data at *IJE* online).

Women who gained >10 kg were more likely to have a younger age at menarche and at first birth, two or more children, lower education and lower body weight at age 20 years, to be older at cohort entry, a former smoker and more likely to have used HRT compared with stable-weight women (±2.5 kg) (Table 1).

There was no association with the risk of premenopausal breast cancer, but there was a positive association of postmenopausal breast cancer in postmenopausal women gaining 5–10 kg (HR = 1.16; 1.02–1.33) and in both women who were pre- (HR = 1.29; 1.07–1.55) and postmenopausal at baseline (HR = 1.33; 1.18–1.50) and gained >10 kg compared with stable-weight women (Table 2).

**Table 2:** Hazard ratio (HR) estimates of breast-cancer risk for weight change from age 20 years to baseline with follow-up on weight as an update in women with breast cancer overall (ca = 6352), pre/perimenopausal (ca = 1461) and postmenopausal breast cancer (ca = 4891) at diagnosis

	Overall (ca = 6352)								Premenopausal (ca = 1461)^c^								Postmenopausal (ca = 4891)^d^							
	Model 1^a^				Model 2^b^				Model 1				Model 2				Model 1				Model 2			
Weight change (kg)	Cases (*n*)	HR	95% CI		Cases (*n*)	HR	95% CI		Cases (*n*)	HR	95% CI		Cases (*n*)	HR	95% CI		Cases (*n*)	HR	95% CI		Cases (*n*)	HR	95% CI	
<–2.5	391	0.93	0.82	1.06	391	0.95	0.83	1.09	135	0.99	0.78	1.24	135	1.03	0.81	1.32	256	0.96	0.80	1.15	256	0.95	0.78	1.15
±2.5	731	1	Ref.		731	1	Ref.		264	1	Ref.		264	1	Ref.		467	1	Ref.		467	1	Ref.	
2.51–5	629	1.10	0.99	1.22	629	1.09	0.98	1.22	191	0.98	0.81	1.18	191	0.98	0.81	1.19	438	1.18	1.01	1.37	438	1.17	1.01	1.37
5.1–10	1421	1.14	1.04	1.25	1421	1.13	1.03	1.24	354	1.02	0.86	1.20	354	1.03	0.87	1.22	1067	1.20	1.05	1.37	1067	1.17	1.02	1.34
10+	3180	1.24	1.14	1.34	3180	1.23	1.13	1.34	517	0.97	0.83	1.13	517	0.99	0.84	1.16	2663	1.37	1.22	1.55	2663	1.36	1.20	1.53
*P*-trend		<0.0001				<0.0001				0.79				0.84				<0.0001				<0.0001		

a Stratified by centre and age at recruitment (categorical 5-year interval).

b Stratified by centre and age at recruitment (categorical 5-year interval) and adjusted for weight at age 20 years (continuous), height (continuous), age at menarche (<12, 12, 13, 14, ≥15 years, missing), number of pregnancies (nulliparous, 1, 2, 3, 4, 5, >5, missing), age at first pregnancy (<20, 20–30, >30 years, nulliparous, missing), education (none, primary school, technical/professional school, secondary school, university degree, missing), alcohol consumption (non-drinkers, ≤1, >1 glass/week, missing), smoking status (never, former, current smoker, missing), physical activity (minimum, moderate, intense, missing), use of oral contraceptives (never, ever users, missing) and menopausal hormone therapy (never, ever users, missing).

c Pre/perimenopausal at cohort entry and premenopausal at diagnosis.

d Pre/perimenopausal or postmenopausal at cohort entry and postmenopausal at diagnosis.

95% CI, 95% confidence interval.

Overall weight change was not associated with risk of premenopausal breast cancer (Table 3). For postmenopausal breast cancer, weight gain was associated with 24% (5–47%) and 42% (22–65%) increased risk in women who were lean at age 20 years who gained 5–10 and >10 kg, respectively, compared with lean women at age 20 years with stable weight.

**Table 3:** Cross-classification of weight change and body mass index (BMI) at age 20 years in overall (*n* = 150 257), pre/peri (*n* = 78 506) and postmenopausal women (71 751)

Overall (*n* = 150 257/ca = 6352)												Pre-/perimenopausal (*n* = 78 506/ca = 1461)											Postmenopausal (*n* = 71 751/ca = 4891)										
	BMI20 <25 kg/m^2^ ca = 3855				BMI20 ≥25 kg/m^2^ ca = 2497d				BMI20 ≥25 kg/m^2^e			BMI20 <25 kg/m^2^ ca = 523				BMI20 ≥25 kg/m^2^ ca = 938d				BMI20 ≥25 kg/m^2^e			BMI20 <25 kg/m^2^ ca = 3332				BMI20 ≥25 kg/m^2^ ca = 1559d				BMI20 ≥25 kg/m^2^e		
Weight change (kg)	Cases (*n*)	HR^a^	95% CI		Cases (*n*)	HR^b^	95% CI		HR	95% CI		Cases (*n*)	HR	95% CI		Cases (*n*)	HR	95% CI		HR	95% CI		Cases (*n*)	HR	95% CI		Cases (*n*)	HR	95% CI		HR	95% CI	
<–2.5	126	0.85	0.68	1.05	265	1.02	0.86	1.21	1.05	0.89	1.24	29	1.09	0.65	1.84	106	0.75	0.51	1.10	1.04	0.77	1.41	97	0.93	0.72	1.21	159	1.03	0.79	1.35	0.96	0.72	1.27
±2.5	351	1.00	Ref.		380	0.97	0.83	1.13	1.00	Ref.		81	1.00	Ref.		183	0.72	0.52	1.00	1.00	Ref		270	1.00	Ref		197	1.08	0.86	1.35	1.00	Ref	
2.51–5	340	1.04	0.89	1.22	289	0.97	0.83	1.15	1.00	0.86	1.17	65	0.93	0.63	1.39	126	0.73	0.53	1.03	1.02	0.80	1.30	275	1.17	0.96	1.42	163	1.25	0.99	1.58	1.16	0.90	1.49
5.1–10	865	1.12	0.99	1.28	556	1.01	0.88	1.16	1.04	0.91	1.19	119	0.74	0.52	1.06	235	0.85	0.63	1.16	1.18	0.96	1.45	746	1.24	1.05	1.47	321	1.08	0.88	1.33	1.00	0.80	1.26
10+	2173	1.26	1.12	1.42	1007	1.14	1.00	1.30	1.18	1.04	1.33	229	0.95	0.69	1.30	288	0.70	0.51	0.94	0.96	0.79	1.18	1944	1.42	1.22	1.65	719	1.30	1.10	1.55	1.21	0.99	1.47
*P*-int^c^									0.48											0.05											0.33		

a HR, hazard ratio.

b Stratified by centre and age at recruitment (categorical 5-year interval) and adjusted for weight at age 20** **years, height, age at menarche, number of pregnancies, age at first pregnancy, education, alcohol consumption, smoking status, physical activity, use of oral contraceptives and menopausal hormone therapy.

c *P*-int = *P* for interaction using likelihood-ratio test comparing cross-classification model (BMI20*weight change) with a model including BMI20 and weight change as separate variables.

d This column has BMI20 <25 and ±2.5 kg as reference group.

e This column has BMI ≥25 and ±2.5 kg as reference group.

95% CI, 95% confidence interval; HR, hazard ratio.

In never HRT users, weight gain >10 kg was associated with increased postmenopausal breast-cancer risk (HR = 1.40; 1.16–1.68) compared with stable weight (Table 4). In HRT ever users, weight gain >10 kg was also associated with an increased breast-cancer risk (HR = 1.23; 1.04–1.44) compared with those with stable weight. In ever HRT users who were lean at age 20 years, weight gain >5 kg was positively associated with risk of postmenopausal breast cancer compared with stable weight (5.1–10 kg; HR = 1.23; 1.01–1.51) (>10 kg; HR = 1.31; 1.08–1.58) (Table 5a). There was no evidence for an association of weight gain with the risk of postmenopausal breast cancer in women overweight at age 20 years when compared with women with stable weight in the same category by BMI at age 20 years and HRT use (Table 5a). Compared with never HRT users with BMI_20_ <25 kg/m² and stable weight, weight gain >10 kg was positively associated with postmenopausal breast-cancer risk in women who were lean (HR = 1.52; 1.24–1.86) or overweight at age 20 years (HR = 1.50; 1.23–1.84) in never HRT users and in all HRT users (Table 5b).

**Table 4:** Cross-classification of weight change and hormone replacement therapy (HRT) use in postmenopausal women (*n* = 66 479)

Postmenopausal (*n* = 66 479/ca = 3409)^c^											
Never HRT users (ca = 1369)					Ever HRT users (ca = 2040)^d^				Ever HRT users^e^		
Weight change (kg)	Cases (*n*)	HR^a^	95% CI		Cases (*n*)	HR	95% CI		HR	95% CI	
<–2.5	88	0.99	0.75	1.32	114	1.55	1.17	2.04	0.99	0.76	1.28
±2.5	134	1.00	Ref.		187	1.57	1.25	1.97	1.00	Ref.	
2.51–5	104	1.07	0.83	1.38	192	1.82	1.45	2.27	1.16	0.94	1.42
5.1–10	256	1.01	0.81	1.25	481	1.85	1.52	2.26	1.18	0.99	1.41
10+	787	1.40	1.16	1.68	1066	1.92	1.60	2.32	1.23	1.04	1.44
*P*-int^b^									<0.0001		

a Stratified by centre and age at recruitment (categorical 5-year interval) and adjusted for weight at age 20 years, height, age at menarche, number of pregnancies, age at first pregnancy, education, alcohol consumption, smoking status, physical activity, use of oral contraceptives and menopausal hormone therapy.

b *P*-int = *P* for interaction using likelihood-ratio test comparing cross-classification model (weight change*HRT) with a model including HRT and weight change as separate variables.

c Out of 71 751 postmenopausal women at cohort entry, there were 66 479 with information on HRT. Of all the 4891 postmenopausal breast-cancer cases, 3409 had information on HRT.

d This column has never HRT users and ±2.5 kg as reference group.

e This column has ever HRT users and ±2.5 kg as reference group.

95% CI, 95% confidence interval; HR, hazard ratio.

**Table 5a:** Cross-classification by weight change, body mass index at age 20 years (BMI20) and hormone replacement therapy (HRT) with the same BMI and HRT status as reference

Postmenopausal (*n* = 66 479/ca = 3409)^b^																
Never HRT users (n = 33 531)									Ever HRT users (n = 32 948)							
	BMI20 <25 kg/m^2^ ca = 939				BMI20 ≥25 kg/m^2^ ca = 430				BMI20 <25 kg/m^2^ ca = 1 412				BMI20 ≥25 kg/m^2^ ca = 628			
Weight change (kg)	Cases (*n*)	HR^a^	95% CI		Cases (*n*)	HR	95% CI		Cases (*n*)	HR	95% CI		Cases (*n*)	HR	95% CI	
<–2.5	28	0.91	0.64	1.30	60	0.88	0.60	1.28	50	0.94	0.68	1.29	64	1.07	0.67	1.73
±2.5	81	1.00	Ref.		53	1.00	Ref.		111	1.00	Ref.		76	1.00	Ref.	
2.51–5	60	1.14	0.88	1.47	44	0.92	0.64	1.32	119	1.16	0.92	1.46	73	1.39	0.91	2.11
5.1–10	183	1.23	0.98	1.54	73	0.81	0.59	1.10	347	1.23	1.01	1.51	134	1.30	0.90	1.86
10+	587	1.52	1.24	1.86	200	1.05	0.80	1.37	785	1.31	1.08	1.58	281	1.33	0.95	1.87

a Stratified by centre and age at recruitment (categorical 5-year interval) and adjusted for weight at age 20 years, height, age at menarche, age at first pregnancy, education, alcohol consumption, smoking status, physical activity and use of oral contraceptives.

b Out of 71 751 postmenopausal women at cohort entry, 66 479 women had informaton on BMI at age 20 years and HRT. Out of 4891 postmenopausal breast-cancer cases, 3409 breast-cancer cases had information on BMI at age 20 years and HRT.

95% CI, 95% confidence interval; HR, hazard ratio.

**Table 5b:** Cross-classification by weight change, body mass index at age 20 years (BMI20) and hormone replacement therapy (HRT)

Postmenopausal (*n* = 66 479/ca = 3409)^b^																
Never HRT users (*n* = 33 531)									Ever HRT users (*n* = 32 948)							
	BMI20 <25 kg/m^2^ ca = 939				BMI20 ≥25 kg/m^2^ ca = 430				BMI20 <25 kg/m^2^ ca = 1 412				BMI20 ≥25 kg/m^2^ ca = 628			
Weight change (kg)	Cases (*n*)	HR^a^	95% CI		Cases (*n*)	HR	95% CI		Cases (*n*)	HR	95% CI		Cases (*n*)	HR	95% CI	
<–2.5	28	0.91	0.64	1.30	60	1.26	0.89	1.79	50	1.59	1.14	2.21	64	1.55	1.03	2.32
±2.5	81	1.00	Ref.		53	1.44	1.05	1.96	111	1.69	1.33	2.16	76	1.44	0.99	2.09
2.51–5	60	1.14	0.88	1.47	44	1.32	0.95	1.82	119	1.96	1.54	2.50	73	2.00	1.44	2.76
5.1–10	183	1.23	0.98	1.54	73	1.16	0.90	1.50	347	2.09	1.69	2.59	134	1.86	1.46	2.38
10+	587	1.52	1.24	1.86	200	1.50	1.23	1.84	785	2.22	1.81	2.71	281	1.91	1.56	2.35

a Stratified by centre and age at recruitment (categorical 5-year interval) and adjusted for weight at age 20** **years, height, age at menarche, age at first pregnancy, education, alcohol consumption, smoking status, physical activity and use of oral contraceptives.

b Out of 71** **751 postmenopausal women at cohort entry, 66** **479 women had informaton on BMI at age 20** **years and HRT. Out of 4891 postmenopausal breast-cancer cases, 3409 breast-cancer cases had information on BMI at age 20** **years and HRT.

95% CI, 95% confidence interval; HR, hazard ratio.

Table 6 shows HR estimates for weight change and breast-cancer risk by hormone-receptor status. Weight gain >10 kg was associated with a 24% increased risk (6–44%) of ER+ and a 46% increased risk (15–85%) of ER+PR+ breast cancer compared with women with stable weight and the same receptor status. Associations with weight gain for hormone-receptor-negative breast-cancer subtypes were similar to those for hormone-receptor-positive subtypes, but estimates were imprecise.

**Table 6:** Hazard ratio (HR) estimates of weight change from age 20 years to baseline and risk of breast cancer overall by hormone-receptor status (*n* = 150** **257)

	ER+^b^ ca = 2190				ER–^c^ ca = 492				ER+PR+^d^ ca = 1062				ER–PR–^e^ ca = 330			
Weight change (kg)	Cases (*n*)	HR^a^	95% CI		Cases (*n*)	HR	95% CI		Cases (*n*)	HR	95% CI		Cases (*n*)	HR	95% CI	
<–2.5	103	0.85	0.66	1.09	29	0.93	0.56	1.54	44	0.82	0.54	1.25	19	0.95	0.49	1.83
±2.5	219	1.00	Ref		49	1.00	Ref		100	1.00	Ref		30	1.00	Ref	
2.51–5	205	1.17	0.97	1.42	47	1.07	0.70	1.62	99	1.69	1.27	2.24	33	1.13	0.66	1.94
5.1–10	454	1.01	0.85	1.20	116	1.32	0.93	1.87	234	1.29	0.99	1.67	79	1.54	0.99	2.40
10+	1209	1.24	1.06	1.44	251	1.26	0.91	1.73	585	1.46	1.15	1.85	169	1.44	0.95	2.18
*P*-trend		<0.0001				0.06				0.002				0.03		

a Stratified by centre and age at recruitment (categorical 5-year interval) and adjusted for weight at age 20** **years, height, age at menarche, age at first pregnancy, education, alcohol consumption, smoking status, physical activity, use of oral contraceptives and hormone replacement therapy.

b ER+ = estrogen-receptor-positive breast cancer.

c ER– = estrogen-receptor-negative breast cancer.

d ER+PR+ = estrogen-and-progesterone-receptor-positive breast cancer.

e ER–PR– = estrogen-and-progesterone-receptor-negative breast cancer.

95% CI, 95% confidence interval.

## Discussion

In this large cohort of women from six European countries, long-term weight gain was not associated with premenopausal breast cancer, but was positively associated with postmenopausal breast-cancer risk overall in women who were lean at age 20 years, and in HRT users and non-HRT users, with the strongest association in non-HRT users who were lean at age 20 years. Weight gain was associated with an increased risk of ER+ and ER+PR+ breast cancer with similar associations for hormone-receptor-negative subtypes.

Compared with the previous EPIC analyses on long-term weight change, the current study had longer follow-up, included more breast-cancer cases and had weight at follow-up in addition to weight at age 20 years and cohort entry. Our finding of a positive association between long-term weight gain and postmenopausal breast cancer is in line with evidence from several studies.^9^^,^^10^^,^^14–17^ An earlier EPIC analysis on weight change from age 20 years to cohort entry with 1358 cases reported that only in non-HRT users was weight gain >15 kg associated with an increased postmenopausal breast-cancer risk compared with stable weight.^10^ The current study observed associations between weight gain and risk of breast cancer in both HRT users and non-HRT users. The effect of weight gain on postmenopausal breast-cancer risk was strongest in non-HRT users in the current study. This finding is in agreement with the results from the previous EPIC analysis on adult weight change and risk of breast cancer.^10^ A major new result with respect to the previous analysis in EPIC is that weight gain was associated with postmenopausal breast cancer in non-HRT users who were lean at age 20 years.

Consistently with our findings, a meta-analysis of 16 studies on weight gain reported a 7% increased risk (95% confidence interval 5–9%) of postmenopausal breast cancer per 5-kg weight gain.^16^ Another cohort study (breast-cancer cases = 900/*n* = 28 153) reported that weight gain before or around menopause was associated with a 38–69% increased breast-cancer risk, but there was no clear risk increase associated with later weight gain.^15^ The National Institutes of Health-AARP Diet and Health Study (breast-cancer cases = 2,111/*n* = 99 039) reported that weight gain from age 18 years to middle adulthood was associated with increased risk of postmenopausal breast cancer in non-HRT users.^18^

In disagreement with our result of no association of long-term weight gain with risk of premenopausal breast cancer, the Premenopausal Breast Cancer Collaborative Group found an inverse association between BMI and premenopausal breast cancer, with the strongest association for BMI at age 18–24 years.^19^ Results were similar in a meta-analysis of 13 studies on adult weight gain reporting that greater early adult BMI was inversely associated with premenopausal breast cancer.^16^ A recent pooled study of 17 prospective cohort studies from the Premenopausal Breast Cancer Collaborative Group found that weight gain from 18–24 to 35–54 years was inversely associated with premenopausal breast cancer (HR per 5 kg = 0.96; 0.95–0.98) and with ER+ breast-cancer risk (HR per 5 kg = 0.96; 0.94–0.98).^20^

An earlier EPIC-PANACEA study on shorter-term weight change in middle adulthood^11^ reported a positive association between high weight gain (0.83–4.98 kg/year) and premenopausal cancer (HR = 1.37; 1.02–1.85) and no association with postmenopausal breast cancer (HR = 1.07; 0.96–1.20).^11^ Further, The Nurses’ Health Study reported that the association of short-term (4-year) weight gain was stronger for premenopausal women (RR 1.38; 1.13–1.69) than for postmenopausal women (RR 1.10; 0.97–1.25).^21^

The inconsistent findings between the current study and the EPIC-PANACEA study indicate a difference between the effect of longer-term and shorter-term weight change on risk of breast cancer. Compared with the current study, the EPIC-PANACEA study looked at weight gain at a later time point in life (women aged 40–50 years). One possible explanation for the contradictory findings could be that weight gain in middle adulthood leads to a different fat deposition than weight gain earlier in life, with more intra-abdominal fat gain in middle adulthood.^22–24^

Our findings for weight gain and risk of ER+ and ER+PR+ breast cancer, as well as ER– and ER–PR– breast cancer, are in line with several findings in other studies reporting no evidence for heterogeneity by hormone-receptor status^11^^,^^16^^,^^25^ and a previous EPIC analysis investigating the relationship between BMI, HRT and breast-cancer risk by age and hormone-receptor status.^26^

Oestrogens are considered to stimulate ductal growth and cell proliferation of breast epithelial cells^27^ and high levels of serum oestrogens for a given age are associated with an increased risk of breast cancer among postmenopausal women^27^^,^^28^ but not among premenopausal women because of the complexity of measuring the cyclic variation of oestrogens.^29^^,^^30^

One possible explanation for our findings could be due to periods of hormonal changes through life (i.e. menarche, pregnancy and menopause) and the different role that body fat plays at different stages. During a menstrual cycle, fat tissue is involved in regulating hormones (oestrogen and progesterone) that make up the menstrual cycle. Premenopausal women with a high BMI have more anovulations, lower oestrogen levels during the anovulatory cycles and lower progesterone levels in the luteal phase than leaner women.^31^ At menopause, surplus fat in the body leads to an excess of plasma levels of oestrogens and low levels of sex-hormone-binding globulin. Subcutaneous fat has higher concentrations of aromatase (the enzyme that converts precursors to estradiol) and therefore high levels of subcutaneous fat are more associated with elevated oestrogens in postmenopausal women than are high levels of visceral fat.

Another possible explanation could be that there are differences in the location of fat deposition before and after menopause that have different effects on the breast.^32^

A major strength of the current study is that weight assessment was standardized. Another strength is the assessment of weight at three different time points and the follow-up information on most of the covariates. A limitation of the study is that data on menopausal status at diagnosis were not available and therefore age at diagnosis was used as a proxy. This could have led to misclassification of menopausal status at breast-cancer diagnosis. Weight assessments at age 20 years and follow-up were self-reported, which could have led to misclassification of weight change. However, validation studies have been done in centres where weight at cohort entry was both self-reported and measured, and showed good agreement between self-reported and measured weight.^33^^,^^34^ It is likely the same for self-reported weight at follow-up in the current study. However, we cannot rule out the possibility of misclassification. As the current study is a prospective cohort study, it is unlikely that this possible misclassification is related to breast-cancer occurrence. Another limitation is the unavailability of data on changes in waist circumference, which could have been a better predictor of breast-cancer risk.^35^

## Conclusion

Our findings show that long-term weight gain was associated with increased postmenopausal breast-cancer risk in women who were lean at age 20 years, HRT users and non-users, and hormone-receptor-positive breast cancer.

## Additional authors

Maria Jose Sánchez^18–21^, Maria Dolores Chirlaque Lopez^22^, Eleni Peppa^23^, Antonia Trichopoulou^23^, Georgia Martimianaki^23^, Antonio Agudo^24^, Carmen Santiuste^25–26^, Eva Ardanaz^20^^,^^27^^,^^28^, Pilar Amiano^20^^,^^29^, Marie-Christine Boutron-Ruault^30^, Vittorio Simeon^31^, Franco Berrino^32^, Rosario Tumino^33^, Gianluca Severi^30^^,^^34^, Tanja Stocks^14^, Renée Turzanski-Fortner^9^, Krasimira Aleksandrova^35^, Charlotta Rylander^13^, Dagfinn Aune^1^^,^^36^^,^^37^ and Christina C Dahm^6^

^18^Escuela Andaluza de Salud Pública (EASP), Granada, Spain, ^19^Instituto de Investigación Biosanitaria ibs. GRANADA, Granada, Spain, ^20^Centro de Investigación Biomédica en Red de Epidemiología y Salud Pública (CIBERESP), Madrid, Spain, ^21^Department of Preventive Medicine and Public Health, University of Granada, Granada, Spain, ^22^Department of Epidemiology, University of Murcia, Murcia, Spain, ^23^Hellenic Health Foundation, Athens, Greece, ^24^Unit of Nutrition and Cancer, Catalan Institute of Oncology—ICO, Nutrition and Cancer Group, Bellvitge Biomedical Research Institute—IDIBELL, L'Hospitalet de Llobregat, Barcelona, Spain, ^25^Department of Epidemiology, Murcia Regional Health Council, IMIB-Arrixaca, Murcia, Spain, ^26^CIBER Epidemiología y Salud Pública (CIBERESP), Spain, ^27^Navarra Public Health Institute, Pamplona, Spain, ^28^IdiSNA, Navarra Institute for Health Research, Pamplona, Spain, ^29^Ministry of Health of the Basque Government, Public Health Division of Gipuzkoa, Biodonostia Health Research Institute, Donostia-San Sebastian, Spain, ^30^Université Paris-Saclay, UVSQ, Univ. Paris-Sud, Inserm, Équipe ‘Exposome, Hérédité, Cancer et Santé’, CESP, Gustave Roussy, 94805, Villejuif, France, ^31^Department Of Mental, Physical Health and Preventive Medicine, Medical Statistics Unit, University of Campania ‘Luigi Vanvitelli’, Naples, Italy, ^32^Association La Grande Via, Villa La Mausolea, Soci, Bibbiena, Arezzo, Italy, ^33^Cancer Registry and Histopathology Department, Provincial Health Authority (ASP), Ragusa, Italy, ^34^Department of Statistics, Computer Science, and Applications ‘G. Parenti’ (DISIA), University of Florence, Italy, ^35^Department of Nutrition and Gerontology, German Institute of Human Nutrition Potsdam-Rehbruecke, Nuthetal, Germany, ^36^Department of Nutrition, Bjørknes University College, Oslo, Norway and ^37^Department of Endocrinology, Morbid Obesity and Preventive Medicine, Oslo University Hospital, Oslo, Norway

## Supplementary data

Supplementary data are available at *IJE* online.

## Funding

The coordination of EPIC is financially supported by the European Commission (DG-SANCO) and the International Agency for Research on Cancer. The national cohorts are supported by the Danish Cancer Society (Kræftens Bekæmpelse) (Denmark), German Cancer Aid (Deutsche Krebshilfe), German Cancer Research Center (Deutsches Krebsforschungszentrum, DKFZ), Federal Ministry of Education and Research (Bundesministerium für Bildung und Forschung, BMBF) (Germany), Associazione Italiana per la Ricerca sul Cancro-AIRC-Italy and National Research Council (Italy), Swedish Cancer Society (Cancerfonden), Swedish Research Council (Vetenskapsrådet), County Councils of Skåne and Västerbotten (Sweden), Cancer Research UK (14136 to EPIC-Norfolk; C570/A16491 and C8221/A19170 to EPIC-Oxford), Medical Research Council (1000143 to EPIC-Norfolk; MR/M012190/1 to EPIC-Oxford) (UK). Infrastructure support for the Department of Epidemiology and Biostatistics at Imperial College London (UK) was provided by the NIHR Imperial Biomedical Research Centre (BRC). The funders had no role in the design and conduct of the study; the collection, analysis and interpretation of the data; or the preparation, review and approval of the manuscript; or in the decision to submit the manuscript for publication.

## Supplementary Material

dyab032_Supplementary_Data
