# Supplementary figures and images for: Long-term weight change and risk of breast cancer in the European Prospective Investigation into Cancer and Nutrition (EPIC) study
Source: Int J Epidemiol. 2021 Mar 23;50(6):1914–26. doi: 10.1093/ije/dyab032 (PMC8743116; doi:10.1093/ije/dyab032)

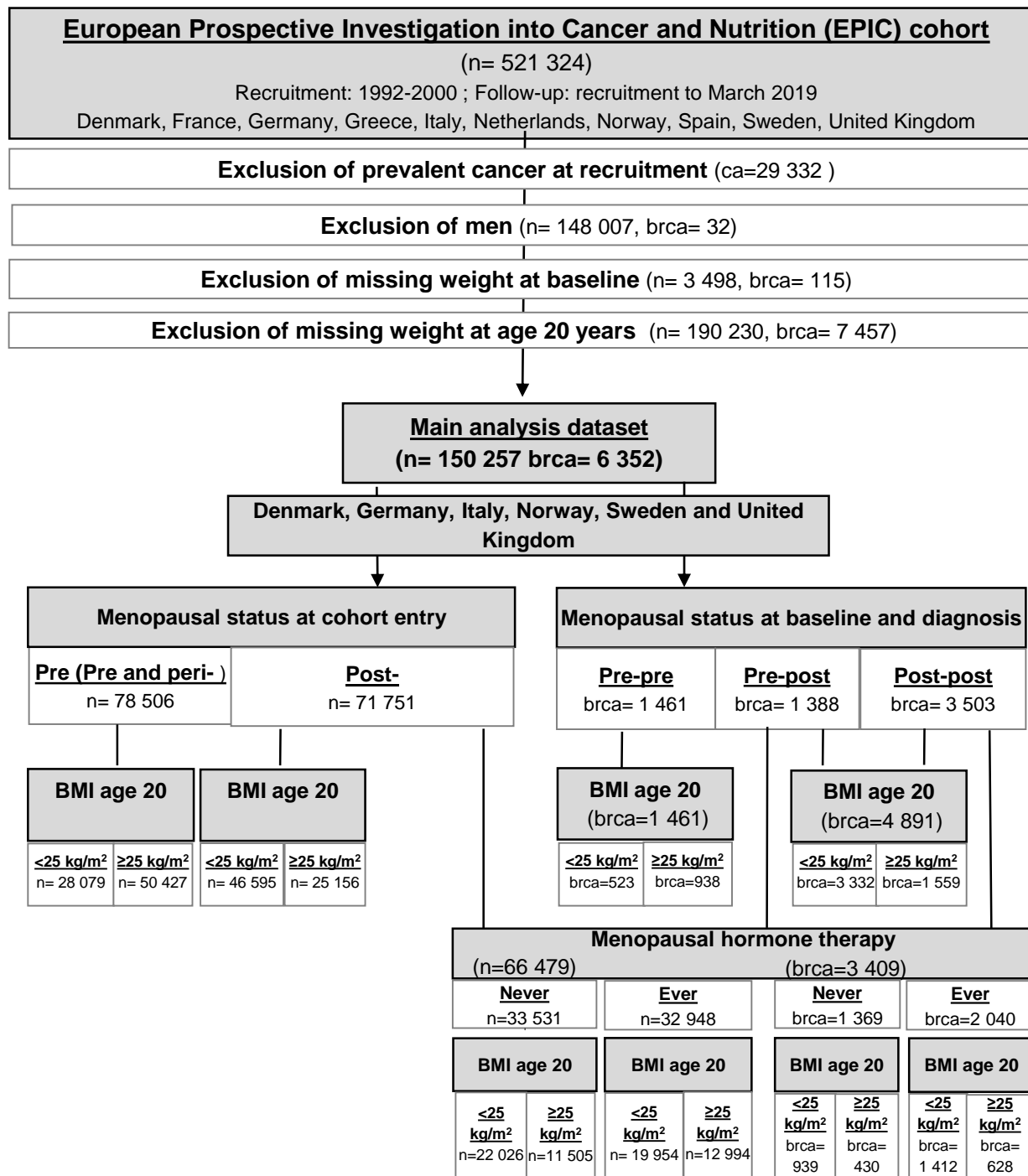

Supplement: dyab032_Supplementary_Data [file dyab032_supplementary_data.zip › ije-2020-06-1210-File009.pdf]
